# Reconceptualized Associative Learning

**DOI:** 10.1007/s40614-025-00442-8

**Published:** 2025-04-02

**Authors:** C. R. Gallistel

**Affiliations:** Rutgers Center for Cognitive Science, 152 Frelinghuysen Road, Piscataway, NJ 08854-8020 USA

**Keywords:** Informativeness, Communicated information, Measure of association, Strength of evidence, Assignment of credit, Change detection, Time allocation

## Abstract

Research on the role of time in associative learning has changed our understanding of what an association is. It is a measurable fact about the distribution of events in time, not an altered activation-conducting connection in a mind, brain or net. Associative learning is the process of perceiving temporal associations and deciding to act on them. Informativeness— the ratio of a conditional rate to an unconditional rate—is the essential empirical variable, not the probability of reinforcement. The communicated information between temporally associated behavioral and reinforcing events is the log of informativeness. Because the time units in the rate estimates cancel, associative-learning is time-scale invariant: Perceivably associated events may be arbitrarily widely separated. There are no windows of associability nor decaying eligibility traces. The learning rate—operationally defined as the reciprocal of reinforcements prior to the appearance of a conditioned response—is an almost scalar function of relative temporal separation, as measured by informativeness. The central role of informativeness unites our understanding of Pavlovian and operant/instrumental phenomena, revealing unexpected quantitative and conceptual communalities.

In 1967, Robert Rescorla reviewed the shortcomings in control procedures then used in Pavlovian conditioning experiments (Rescorla, 1967). He pointed out that they replaced one contingency with another. He stressed that in a proper control, “the CS provides *no information* about subsequent occurrences of the US” which means that the distributions of CSs and USs “must be such that the CS occurrences do not predict the occurrence of the USs at *any* time . . .” no matter how remote (p. 74; emphasis in original).

He argued for the truly random control in which reinforcements are programmed by Poisson processes, which makes them equally likely at every moment in time. He then used that control to show that associative learning depended on the contingency between the conditional stimulus (CS) and reinforcement (R), not on their temporal pairing (Rescorla, 1968, 1969; Rescorla & Skucy, 1969). He pointed out that “. . . the yoked control [in instrumental conditioning] was introduced precisely to determine what effects are uniquely due to instrumental reinforcement contingencies.”

Rescorla remarked that the contingency was a function of the proportions of USs (hereafter denoted by R’s for reinforcements) that occurred during the CSs and in its absence. However, he incorrectly assumed that these proportions could be reduced to two probabilities, the probability of R occurring during the CS and the probability of its occurring in its absence. In equating proportions with probabilities, he failed to distinguish between rate and probability. Over the last half century, we have learned that the distinction is fundamental. His deep insight into the nature of associative learning can only be realized by shifting our attention from the probability of reinforcement to the rate of reinforcement.

A probability is the proportion between a count of the “successes” (the Rs) divided by the sum of successes and “failures” (the $\sim \text{R}$’s):

$${p}_{\text{R}}={n}_{\text{R}}/\left({n}_{\text{R}}+{n}_{\sim \text{R}}\right)$$

A rate is a count divided by the duration ($T$) of the interval over which the count is made:

$${\lambda}_{\text{R}}={n}_{\text{R}}/T$$

The failure to distinguish between probability and rate was and remains common. It arises in part from the traditional conception of what an association is, psychological speaking. That conception has roots in the philosophy of mind that go back to Aristotle. In philosophy, psychology, behaviorist cognitive science, and neuroscience, associations reside in minds, hence in brains. Their function is to conduct an activating or deactivating signal from one idea to another, or from one node to another, or from one neuron to another. It is the concept of the plastic synapse (Hebb, 1949). Central to this conception is that reinforcements strengthen an activating connection (a positive association) and nonreinforcements have the opposite effect; they weaken a positive association and/or strengthen a negative association. This conception is explicit in the Rescorla-Wagner model and its many descendants (Kang et al., 2024; Rescorla & Wagner, 1972), that is, in any model that uses delta-rule updating:

1 $$\Delta {A}_{i}R=\alpha \left(\Lambda \left(\ddot{R}\right)-\sum_{i=1}^{i=n}{A}_{i}R\right)$$

Equation (1) is found in some form in every textbook on associative learning and in every review of reinforcement learning. It may be read as follows: The change in the association between a reinforcer, $R$, and the ${i}^{\text{th}}$ conditional stimulus (${A}_{i}$) on any given trial is proportional to the learning rate, $\alpha$, times the difference between $\Lambda \left(\ddot{R}\right)$ and the sum over all of the associations between R and the CSs present on that trial. $\Lambda \left(\ddot{R}\right)$ is the upper limit on the strength of an association to R. $\ddot{R}$ has value, 1 on a trial where R occurs, in which case $\Lambda \left(\ddot{R}\right)\ne 0$. On $\sim \text{R}$ trials, $\Lambda \left(\ddot{R}\right)=0$, which makes $\Delta {A}_{i}R$ negative, which causes positive associations to be decremented and negative associations to be made stronger.

There is a conceptual problem with this formulation closely connected to the assumption that associative learning depends on the probability of reinforcement. The problem was already there in the Hullian model on which Rescorla and Wagner (1972) based their own model. It comes from attributing causal efficacy to $\sim \text{R}$ “events.” Nonreinforcements have no physical attributes. They cannot excite sensors. Events with causal effects must happen at specifiable times. A failure can cause something to happen only if there is a specified time at which a success was expected. There are many circumstances in which there are no such times. When reinforcements are randomly distributed in time, as they are when scheduled by a Poisson process, there are no such times because a reinforcement is equally likely at every moment in time (Gallistel, 2021b). The need to attribute causal efficacy to events that cannot be localized in time explains why the Hullian model and its delta-rule descendants could not and still cannot explain the quantitative facts about extinction (Dayan & Niv, 2008; Gleitman et al., 1954; Kang et al., 2024; Kimble, 1961).

The problem became apparent in Rescorla and Wagner’s (1972) article when they tried to apply Eq. (1) to Rescorla’s contingency-not-pairing results (Fig. 1). Here is how they tried to circumvent it in their simulation: “. . . to exemplify the application of the model to this particular case, the experimental session was taken to be divisible into time segments the length of the CS duration. Each segment containing the CS is thus treated as an AX “trial” [context+CS] and each segment not containing the CS as an A “trial. [context only] It is possible then to specify the sequence of reinforcement and nonreinforcement over each of the two kinds of trials” (Rescorla & Wagner, 1972, p. 88; scare quotes in the original).

**Fig. 1.**
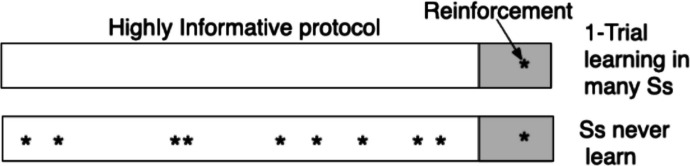
Time lines for the two key protocols in Rescorla (1968). *Note. *Upper protocol is for the “experimental” group. Lower protocol is for the truly random control group (the group of principal interest). The durations of the interval-trial intervals when the noise CS was not present (white) varied about a mean much greater than the fixed duration 2-minute intervals when it was present (gray). The question to ask oneself when looking at the Highly Informative protocol is, How many nonreinforcements were there in the intertrial interval and where did they happen?

In their simulation, they updated the two associative strengths—one for the association between CS and R, the other for the association between context and R—once and only once on each imaginary “trial.” When the Poisson process was running, they assumed that there was one reinforcement on 40% of the “trials” (imagined 2-min intervals) and no reinforcements on 60% of them. On reinforced “trials,” they set $\Lambda \left(\ddot{R}\right)=1$ and incremented the associations between both the context and the CS in accord with Eq. (1). On unreinforced “trials,” they set $\Lambda \left(\ddot{R}\right)=0$ and decremented both ${\text{A}}_{\text{cs}}\text{R}$ and ${\text{A}}_{\text{c}}\text{R}$ when the CS was present but only ${\text{A}}_{\text{c}}\text{R}$ when the CS was absent. The decrementing of the association between R and context on the many imaginary trials that occurred during the ITIs in the upper protocol was the key to explaining why the subjects in that group were much more afraid of the noise (gray) than they were of the context (white).

It is often remarked that the trouble with simulations is that you are doomed to succeed. Success comes eventually because one works around certain “minor” details that seem unimportant but are in fact fatal. The fatal detail here is that Rescorla did not vary the probability of reinforcement, he varied the rate. In the two protocols in Fig. 1, the rate was 0.26 shocks/minute. In any 2-min interval, there were anywhere from 0 to 3 shocks (Gallistel, 2021b). The delta-rule updating model in Eq. (1) is not physically realizable when there are no actual trials, which is to say, during the ITIs when only the background is present. That is why Rescorla and Wagner (1972) put scare quotes around the 2-min “trials” they had to imagine to make the average*s* in their simulation come out right. No partitioning of protocols into “trials” of equal duration will make the model work when given the randomly distributed reinforcements the rats in fact experienced, because the R count does not change as one makes the imaginary “trials” shorter and shorter but the ~R count goes to infinity. As the count of the imagined ~R’s goes to infinity, all probabilities—and therefore all associative strengths—go to 0.

Over the 4 decades since the Rescorla-Wagner delta-rule updating model launched the modern era in the formalization of associative learning (Esber et al., 2025), it has slowly become apparent that the key to a successful formalization is to focus on the rate of reinforcement not the probability. The change in focus has led to a change in the ontological status of an association in psychology, cognitive science, and neuroscience. It’s no longer something in the head; it’s a measurable statistical fact out there in the world, a distal stimulus, not a percept. What is in the head is a percept that represents the strength of the statistical association out there in the world. I here explain the computations by which the percept is thought to be generated and the computations by which it produces measured behavior.

In his 1967 review, Rescorla demurred when it came to specifying a formula for computing contingency. He had good reason to do so because there was then no formula for computing it in continuous time (Gibbon, 1981; Gibbon et al., 1974). Now there is (Balsam et al., 2006; Balsam & Gallistel, 2009). The key to finding a formula was to turn to information theory. Rescorla would have approved because his review stressed the fact that reinforcement was contingent on a CS just in case it communicated information about where the reinforcement was to be found in time. Information theory provides the mathematical foundation for our understanding of what information is and how it is communicated (Cover & Thomas, 1991; Shannon, 1948).

## Measuring Communicated Information

Rescorla realized that to have a quantitative theory of associative learning, we must measure the distal stimulus, the amount of information communicated. We need to measure it as soon as there is anything to measure, which means after the first reinforcement. Probability of reinforcement is useless then, because when the reinforcement occurs when both the CS and its context are present, the probability of reinforcement is 1 for both predictors. If probability drove associative learning, learning to respond to the CS after its first reinforcement (one-shot learning) would be impossible. Learning would have to progress by small changes, which is sometimes taken as definitional (Richards & Kording, 2023). However, one-shot associative learning is seen in both Pavlovian and operant protocols when the statistical association is sufficiently strong (Gallistel & Shahan, 2024; Harris & Gallistel, 2024; Jenkins et al., 1981; Revusky & Garcia, 1970).

An extreme example helps us to intuit why one-shot associative learning should be possible. Suppose a subject has been in a test chamber for 6 hr when a noise comes on and 1 s later they get shocked. We and the subject would be inclined to think the noise was associated with the shock. If someone argued it was “just a coincidence,” most of us would be inclined to respond, “That’s one hell of a coincidence!” The information theoretic computation of the association and its reliability given an *n* of one justify our intuition because they take interval durations into account. The deep problem with probability of reinforcement is that it does not take duration into account.

The information-theoretic measure of communicated information is the log of the ratio of two rates. The rates are counts divided by durations. There are 21,600 s in 6 hr. In the above example, the contextual rate of shock is ${\lambda }_{\text{R}|\text{C}}=1/\text{21,600s}$, and the rate during the 1-s CS is, ${\lambda }_{\text{R}|\text{CS}}=1/1\text{s}$. The ratio of the rate of reinforcement conditional on a CS and the rate in the context in which the CS and the reinforcement occur is the informativeness of their temporal relationship. It is denoted by lower case iota ($\iota$). In the example at hand $\iota ={\lambda}_{\text{R}|\text{CS}}/{\lambda }_{\text{R}|\text{C}}=1/\frac{1}{21600}=21600$. The log to the base 2 of this ratio is 14.4 bits, which, in our example, is the amount of information communicated by the CS about when to expect the next shock.

If a receiver assumes that shocks are randomly distributed within the context in which the shock has occurred—the simplest assumption—then the receiver has considerable uncertainty about when to expect the next shock (somewhere in 6 hr). When the noise comes on, it greatly reduces the uncertainty, because it predicts the shock is imminent (within the next second).

Uncertainty in information theory is measured by the entropy, $H$, of a random variable’s distribution, which is the mean surprisal:

$$H=\sum p\text{log}\left(1/p\right)$$

The amount by which a communicating signal (the noise) can reduce contextual uncertainty (about when to expect shocks) is the communicated information.

The (differential) entropy of the exponential distribution is $H=1-\text{ln}\lambda=1-\text{ln}\left(1/\mu \right)$, where $\uplambda$ is the rate parameter of the exponential distribution and $\mu$ is its reciprocal, the mean interval (also called the time constant). The difference between the entropy of the contextual distribution of intershock intervals and the entropy of the distribution communicated by the noise is the log of the noise’s informativeness:

2 $$\Delta H=\left(1-\text{ln}{\lambda }_{\text{R}|\text{C}}\right)-\left(1-{\text{ln}\lambda}_{\text{R}|\text{CS}}\right)=\text{ln}\frac{{\lambda }_{\text{R}|\text{CS}}}{{\lambda}_{\text{R}|\text{C}}}=\text{ln}\frac{{\mu}_{\text{R}\leftrightarrow \text{R}|\text{C}}}{{\mu }_{\text{R}\leftrightarrow \text{R}|\text{CS}}}=\text{ln}\left(\iota \right)$$

Equation (2) is the formula that eluded Rescorla because the communicated information divided by the available information is the contingency (Gallistel & Latham, 2022). The available information is the amount that removes all uncertainty. It is communicated by a CS or a response (r) only when they and the R occur simultaneously. In the most common Pavlovian protocol, delay conditioning, reinforcement coincides with CS termination on every trial. In that case, the contingency between CS termination and reinforcement is 1. It is, however, useless as a warning because the offset signal comes too late for the subject to make an anticipatory response. The useful warning comes only from CS onset, provided the delay between onset and the shock is long enough for the subject to make a shock-anticipatory response, like freezing or blinking the eye.

For most purposes, what matters in what follows is not the contingency but rather the communicated information. It measures the strength of the stochastic association.

## What is in the Head

In the head are the percepts: the percepts of rates of reinforcement, informativeness, associations and their reliability. Percepts are produced by the computations that extract them from first-order sensory signals. The percept of a color, for example, is a 3-dimensional vector with signed scalar elements (Grassmann, 1853). The percept of a face is a 50-dimensional vector, also with signed scalar elements (Chang et al., 2021; Chang & Tsao, 2017). These neurobiological vectors are not the distal stimuli to which they correspond. The percepts relevant to associative learning are extracted from counts and durations, neither of which is a sensory event. Counts require counting and durations require timing. Both are themselves elementary computational operations. Gallistel (1990) reviewed the already extensive experimental evidence that much learned behavior depends on learned counts, learned intervals, and on rates, which are counts/durations. The literature has grown much larger in the interim.

**Fig. 2:**
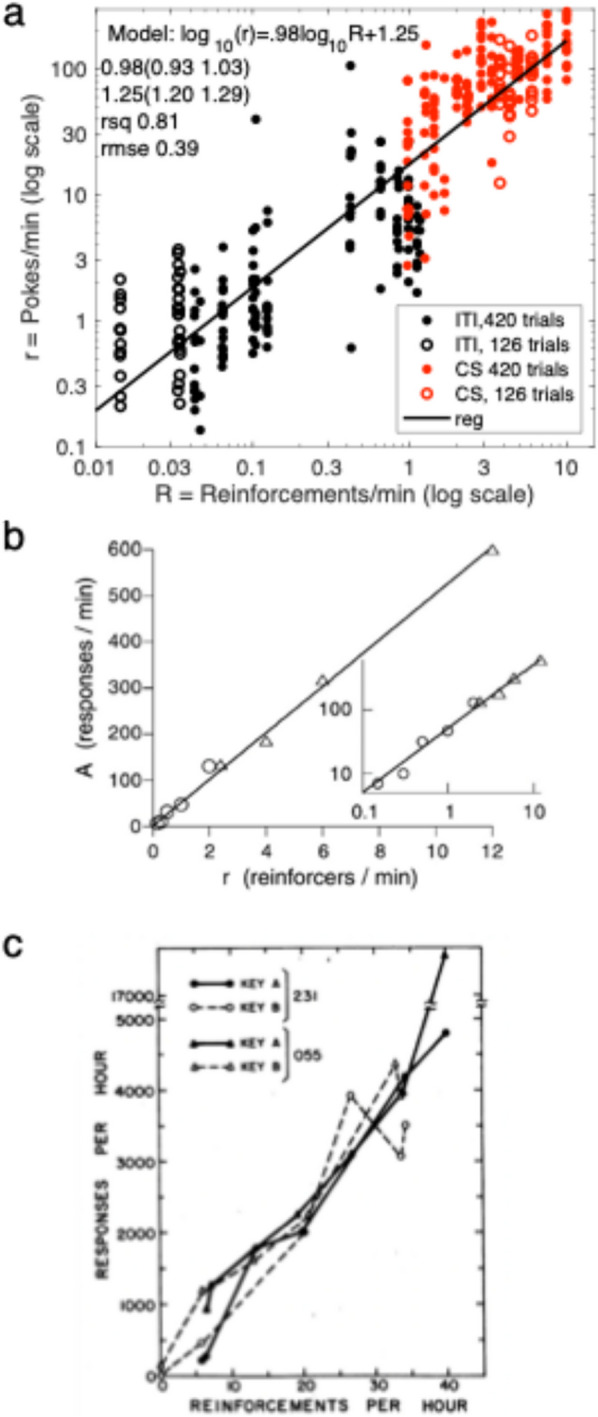
**a.** Hopper entry rate as a function of expected reinforcement rate in a Pavlovian delay-conditioning protocol on double-logarithmic coordinates. The red dots are for pokes during CSs plotted against the expected CS reinforcement rate. The black dots are poke rates during the never-reinforced intertrial intervals plotted against the reinforcement rate expected when in the test chamber (the contextual reinforcement rate). The scales span three orders of magnitude on both axes (reproduced from Figure 5 in Harris & Gallistel, 2024 by permission of the authors). **b**. The rate of adjunctive (unreinforced) floor scratching during intervals when reinforcement was not accessible plotted against the contextual rate of reinforcement in an operant protocol. (Reproduced from Killeen & Sitomer, 2003, Fig. 7, p. 52 by permission of authors and publisher). c. Key pecking rates plotted against rates of reinforcement on concurrent variable interval schedules of reinforcement (Reproduced from Herrnstein, 1961, Fig 2, p. 268 by permission of publisher)

There is now strong experimental evidence that perceived rates of reinforcement are scalar functions of the measured rates of reinforcement, because, under easily arranged circumstances, the function that maps from measured rates of reinforcement to measured rates of responding is scalar over many orders of magnitude (Fig. 2). The scalar relation between measured reinforcement rate and measured response rate implies that the computations that produce percepts of rates of reinforcement are simpler than for either colors or faces:

$${\widehat{\lambda }}_{\text{R}|\text{CS}}={\widehat{n}}_{\text{R}|\text{CS}}/{\widehat{T}}_{\text{CS}}\approx {n}_{\text{R}|\text{CS}}/{T}_{\text{CS}}$$

where and *n* and *T* denote cumulative count and cumulative duration. Variables with hats denote values for percepts in the head, where they are not directly measurable. The variables without hats are directly measured by counting and timing.

The rate of hopper entry during CSs signaling food reinforcement is a scalar function of the CS reinforcement rate (red data points in Fig. 2a). The rate of hopper entry during the never-reinforced intertrial intervals is a scalar function of the contextual rate of reinforcement present (black dots in Figs. 2a). The contextual rate is the rate a subject expects simply from being in the test chamber, without regard to whether the CS is or is not present. The scale factor, $k$, is the same in both cases, $k\cong 18$.

A scalar relation spanning orders of magnitude between rate of responding and rate of reinforcement is evidence that brains compute and remember rates of reinforcement from experienced reinforcements separated in time by arbitrarily long intervals. The argument for this conclusion comes from measurement theory; it rests on two well-established experimental facts:

- (1) rates of responding are proportional to the rates reinforcement (Fig. 2) and;
- (2) the rates of learning are proportional to the ratios of rates of reinforcement (Fig. 4). Together, these results imply a ratio-scale representation of rate of reinforcement, a representation on which all the basic arithmetic operations are valid (Krantz et al., 1971; Krantz, 1972; Luce et al., 1990).

The computation of a rate implies a temporal map: when the first reinforcement in a context occurs, the brain must look back in time to compute the time so far spent in that context because the duration of its experience of the context is the denominator of the rate:

$${\lambda }_{\text{R}|\text{C}}=1/{\mu }_{\text{R}\leftrightarrow \text{R}|\text{C}}$$

In Pavlovian and operant protocols, the context is the test chamber. Subjects may spend hours there prior to the first reinforcement (context habituation). In a Pavlovian protocol with partial reinforcement of the CSs, subjects must compute the cumulative duration of the CSs prior to the first reinforcement in order to compute the CS rate of reinforcement:

$${\lambda }_{\text{R}|\text{CS}}=1/{\mu }_{\text{R}\leftrightarrow \text{R}|\text{CS}}$$

In a reinforcement learning protocol with a long delay between response and reinforcement (Gallistel & Shahan, 2024; Lett, 1975), the subject must also look back to the record of its most recent response to compute the prospective and retrospective informativeness ratios ($\iota$):

$$\overrightarrow{\iota }={\lambda }_{\text{R}|\overrightarrow{\text{r}}}/{\lambda }_{\text{R}|\text{C}}$$

 and

$$\overleftarrow{\iota }={\lambda }_{\text{r}|\overleftarrow{\text{R}}}/{\lambda }_{\text{r}|\text{C}}$$

The first informativeness ratio is the rate of reinforcement looking forward from a response divided by the contextual rate of reinforcement; the second is the rate of responding estimated by looking back from a reinforcement to the most recent response divided by the contextual rate of responding.

The temporal map is a time-stamped record of past events (Arcediano et al., 2003; Balsam & Gallistel, 2009; Cammaerts & Cammaerts, 2022; Chandran & Thorwart, 2021; Eichenbaum & Fortin, 2003; Honig, 1981; Oprisan et al., 2018; Polyanskaya, 2022; Sawa & Kurihara, 2014; Taylor et al., 2014). The raw data record in many contemporary animal-learning labs instantiates the concept. It is a two-column data structure with time or duration stamps in the first column and event codes in the second. Time stamps record the time as given by the computer’s clock. Duration stamps record the intervals between successive events. The time as given by a computer clock (a clock that knows nothing about days, weeks, or months, etc.) may be computed by cumsumming the interevent intervals.

A brain may obtain cumulative elapsed time from a short-period oscillator that increments a counter at the completion of each cycle. Interval durations may be measured using either an array of processes that decay exponentially (Cruzado et al., 2019; Howard, 2018; Howard & Hasselmo, 2020) or as phase differences in arrays of oscillators with different periods (Gallistel, 1990, 2017a). The temporal map is updated event by event. Aristotelean associations don’t get updated in this model, because they do not exist.

The computations that mediate the perception of stochastic associations operate on the data in the map. The map makes possible looking back in time. Humans constantly use their memory to look back in time. So do nonhuman animals (Crystal, 2021)—as will be stressed in the section on the time-scale invariant Learning Rate Law.

## How Associations are Perceived

The computation that maps the measured association out there in the world to the corresponding percept is simple, explicit, and devoid of free parameters. Consider a basic Pavlovian delay protocol like the one whose timeline is shown in the upper part of Fig. 1. The perceived association between the CS and reinforcement may be computed by:

3a $$\widehat{A}\left(\text{CS},\text{R}\right)=\text{log}\left(\widehat{\iota }\right)=\text{log}\frac{{\widehat{\lambda }}_{\text{R}|\text{CS}}}{{\widehat{\lambda }}_{\text{R}|\text{C}}}\approx \text{log}\frac{{\lambda }_{\text{R}|\text{CS}}}{{\lambda }_{\text{R}|\text{C}}}$$

In words, the perceived association, $\widehat{A}\left(\text{CS},\text{R}\right)$, is the log of the perceived informativeness, which is the log of the perceived rate of reinforcement during the CSs divided by the perceived rate of reinforcement in the experimental context. The perceived variables, the ones with hats, are approximately equal to the hatless variables, which we can measure because they are publicly observable. In measurement theory terminology, Eq. (3a) asserts that brains have a ratio-scale representation of the informativeness of the prospective relation between CS onset and rate of reinforcement. The percept is approximately equal to the log of that ratio. It represents the amount of information that CS onset communicates about the expected wait for the next reinforcement.

For an operant example, consider the prospective association between a response and reinforcement when subjects respond on a variable interval (VI) schedule. A VI schedule sets up the next reinforcement at exponentially distributed intervals following the harvesting of the previous reinforcement. The prospective association is the log of the prospective informativeness of a response. The informativeness is the factor by which the response reduces the expected wait for the next reinforcement:

3b $$\widehat{A}\left(\overrightarrow{\text{r}},\text{R}\right)=\text{log}\frac{{\widehat{\mu }}_{\text{R}|\text{C}}}{{\widehat{\mu }}_{\text{R}|\overrightarrow{\text{r}}}}\approx \text{log}\frac{{\mu }_{\text{R}\leftrightarrow \text{R}}}{{\mu }_{\text{r}\to \text{R}}}=\text{log}\left(\overrightarrow{\iota }\right)$$

Or, parameterizing the exponential by rate rather than by mean:

3b’ $$\widehat{A}\left(\overrightarrow{\text{r}},\text{R}\right)=\text{log}\frac{{\widehat{\lambda }}_{\text{R}|\overrightarrow{\text{r}}}}{{\widehat{\lambda }}_{\text{R}|\text{C}}}\approx \text{log}\frac{{\lambda }_{\text{R}|\overrightarrow{\text{r}}}}{{\lambda }_{\text{R}|\text{C}}}=\text{log}\left(\overrightarrow{\iota }\right)$$

**Fig. 3:**
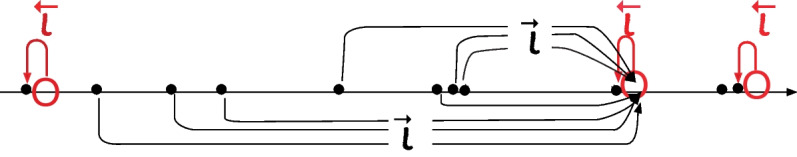
Prospective and Retrospective Informativeness when Subjects Respond on VI Schedules. *Note. *The black dots mark responses, the red open circles mark reinforcements. Black arrows are intervals looking forward from a response to the next reinforcement; red arrows are intervals looking back from a reinforcement to the most recent response

In Eq. (3b), ${\mu }_{\text{R}\leftrightarrow \text{R}}$ is the average interval between reinforcements, and ${\mu }_{\text{r}\to \text{R}}$ is the average interval looking forward from each response to the next reinforcement (see Fig. 3).

Variable interval schedules sustain high and steady rates of responding. Responses are much more frequent than reinforcements (Fig. 3). To see if a response predicts a reinforcement, the brain looks forward in time (black arrows in Fig. 3). It compares the average of these forward intervals to the average interval between reinforcements (intervals between the open red circles). If the average interval looking forward from a response to the next reinforcement is substantially shorter than the average interval between reinforcements, then a response communicates information about where to find the next reinforcement.

To see if a reinforcement retrodicts a response, the brain looks back from reinforcements to the most recent response (red arrows in Fig. 3). If the average interval looking back is substantially shorter than the average interval between responses, then a reinforcement communicates information about where to find the most recent response. The equation for the retrospective association between reinforcement and response is:

3c $$\widehat{A}\left(\text{r},\overleftarrow{\text{R}}\right)=\text{log}\frac{{\widehat{\mu }}_{\text{r}\leftrightarrow \text{r}}}{{\widehat{\mu }}_{\text{r}|\overleftarrow{\text{R}}}}\approx \text{log}\frac{{\mu }_{\text{r}\leftrightarrow \text{r}}}{{\mu }_{\text{r}\leftarrow \text{R}}}=\text{log}\left(\overleftarrow{\iota }\right)$$

Or, again parameterizing by rate rather than by mean:

3c’ $$\widehat{A}\left(\text{r},\overleftarrow{\text{R}}\right)=\text{log}\frac{{\widehat{\lambda }}_{\text{r}|\overleftarrow{\text{R}}}}{{\widehat{\lambda }}_{\text{r}}}\approx \text{log}\frac{{\lambda }_{\text{r}|\overleftarrow{\text{R}}}}{{\lambda }_{\text{r}}}=\text{log}\left(\overleftarrow{\iota }\right)$$

The retrospective contingency was 1 in the control conditions of the Gallistel et al. (2019) experiments with pigeons pecking on VI schedules, because a reinforcement and the peck that triggered it coincided in time. They had the same time stamp to within 0.01 s. When one found the stamp for a reinforcement in the data record, there was no uncertainty about where to find the stamp for the most recent response. The reinforcement communicated all the available information about response recency. The prospective contingency was 0; making a response communicated no information about when to expect the next reinforcement.

The role of retrospective contingency in this rate-based model of reinforcement learning captures the intuition that reinforcements act backward in time in reinforcement learning (Timberlake, 1995). The temporal map also enables the brain of a rat to look back from its first experience of nausea, induced by radiation delivered hours after it experienced a novel taste (Revusky & Garcia, 1970). The rats' in the Revusky and Garcia radiation experiments first experience of nausea occurred when they were months post weanling. The context was their post-weanling experience in the laboratory. The interval from the nausea back to the novel taste was very small in comparison to the preceding months without nausea (the comparison interval). One-shot taste-aversion learning with hours-long delays between taste and nausea is explained without fiddling the parameters of an hypothesized window of association (Logue, 1979). Poison-avoidance learning is simply another manifestation of the time-scale invariance of associative learning. What matters are the relative latencies, not their absolute values.

## Sample Size and Reliability

The first reinforcement in a Pavlovian protocol makes possible the perception of an association between the CS and reinforcement because it defines two rates, the conditional rate and the contextual rate. The same is true for the first reinforcement in an operant protocol; two relevant rates are immediately defined: the response rate in that context and the response rate estimated by looking back from reinforcements to the most recent response. Probabilities are not yet defined when there has been only one reinforcement and neither is correlation, which is the conventional measure of stochastic association. There is, however, the obvious issue of the extent to which estimates of stochastic parameters obtained from a sample of size 1 may be relied on.

An information-theoretic measure of reliability, the *n*D_KL_ statistic, can be computed with a sample size of 1. This computation allows us to judge when one-shot learning is justified by strong evidence. The same computation is assumed to explain the circumstances under which brains draw the same conclusion.

The *n*D_KL_ statistic is the Kullback-Leibler *divergence* (denoted by D_KL_) of the conditional distribution from the unconditional distribution multiplied by the effective sample size (the *n* in *n*D_KL_):

4 $${n\text{D}}_{\text{KL}}\left({\lambda }_{\text{R}|\text{CS}}||{\lambda }_{\text{R}|\text{C}}\right)=\left(\frac{{n}_{\text{R}|\text{CS}}}{1+{n}_{\text{R}|\text{CS}}/{n}_{\text{R}|\text{C}}}\right)\left(\text{ln}\frac{{\lambda }_{\text{R}|\text{CS}}}{{\lambda }_{\text{R}|\text{C}}}+\frac{{\lambda }_{\text{R}|\text{C}}}{{\lambda }_{\text{R}|\text{CS}}}-1\right)$$

The effective sample size is the first expression in parentheses in the middle of Eq. (4); it is a function of the reinforcement counts. In the case we are considering, both counts are 1, so the effective sample size is 0.5. The Kullback-Leibler divergence is the expression enclosed in the second parentheses. The first term in the divergence is the log of the informativeness and the second term is the reciprocal of the informativeness. In the example, we have been considering (21,600s in the context and 1s of noise before the shock), Equation (4) evaluates to 4.5 nats. (The nat is the unit of information when the base of the logarithm is e rather than 10 or 2.)

When there is no divergence, that is, when the informativeness ratio is 1, Peter Latham (Gallistel & Latham, 2022) proved that the ${n\text{D}}_{\text{KL}}$ statistic is distributed gamma(.5,1), which allows us to compute a corresponding *p* value: *p=*1-gamcdf(4.5,.5,1) = .0027. The odds are 370:1 against the hypothesis that the association between the noise CS and shock is just a coincidence.

The *n*D_KL_ statistic is crudely analogous to a *t* test, which is the variance normalized difference between the means of two samples scaled by the square root of the sample size. It is superior in several respects. First, it applies when *n* = 1. Second, the *divergence* between distributions is asymmetric—${n\text{D}}_{\text{KL}}\left({\lambda }_{\text{R}|\text{CS}}||{\lambda }_{\text{R}|\text{C}}\right)\ne {n\text{D}}_{\text{KL}}\left({\lambda }_{\text{R}|\text{C}}||{\lambda }_{\text{R}|\text{CS}}\right)$—whereas a *difference* between two means is symmetric. The asymmetry in a divergence captures the fact that it takes more data to detect a divergence in one direction than it does to detect it when it is in the other direction (Kheifets & Gallistel, 2012). The conventional test does not capture this fact.

The divergence has physical and neurobiological implications not possessed by a *p* value. When converted from nats to bits, the divergence is the average number of additional bits of memory required to encode the intervals from the conditional distribution on the erroneous assumption that they come from the contextual distribution (Cover & Thomas, 1991). This property of the divergence links the new conception closely to the notion that the purpose of associative learning is prediction. In the literature on the information-theoretic approach to stochastic model selection, there is a theorem to the effect that the model that best encodes the data already seen best predicts the data not yet seen when proper account is taken of model complexity (Grünwald, 2005, Section 2.10).

The ability to predict data not yet seen depends on a brain’s statistical model. When the rate predicted by the CS is greater than the rate predicted by the context, the brain has a poor model when it uses its representation of the contextual distribution to predict what will happen during CSs. The Kullback-Leibler divergence measures how poor the contextual model is. The brain reduces memory load and improves its ability to predict the next reinforcement when it switches to a better model of the process generating the reinforcements during CSs.

A final reason to stress the fundamental role of informativeness in associative learning is that it determines the learning rate, when operationally defined as the reciprocal of reinforcements to acquisition. Strong evidence for this empirical law was first published more than 40 years ago. For some reason, it is rarely if ever mentioned in formalizations of the Aristotelean theory of associative learning.

## The Learning Rate Law

In the late 1970s, the Gibbon lab and the Jenkins lab investigated the effect of varying the interval between CSs (the intertrial interval or ITI for short) on the rate of learning. The ITI had been largely ignored on the assumption that it was irrelevant because nothing happens during that interval in most protocols. It turns out, however, that the duration of the ITI has a dramatic effect on the learning rate. Jenkins and his co-authors wrote in the opening of their paper in the same volume: “The effect of trial spacing is so large that no theory of autoshaping [a form of Pavlovian conditioning] can be considered adequate unless it provides an account of how spacing exerts its effects” (Jenkins et al., 1981 p. 255).

The Gibbon lab varied both the ITI duration and the CS duration and discovered that what mattered was not the duration of either by itself but rather the ratio of the cycle duration to the trial duration (Gibbon et al., 1977)—the ratio now called the informativeness. When reinforcements occur only during the CSs, that ratio equals the ratio of the average cycle duration, ${\mu }_{\text{R}\leftrightarrow \text{R}|\text{C}}$ to the average CS duration ${\mu }_{\text{R}\leftrightarrow \text{R}|\text{CS}}$. Gibbon and Balsam (1981) termed this the *C/T* ratio.

**Fig. 4:**
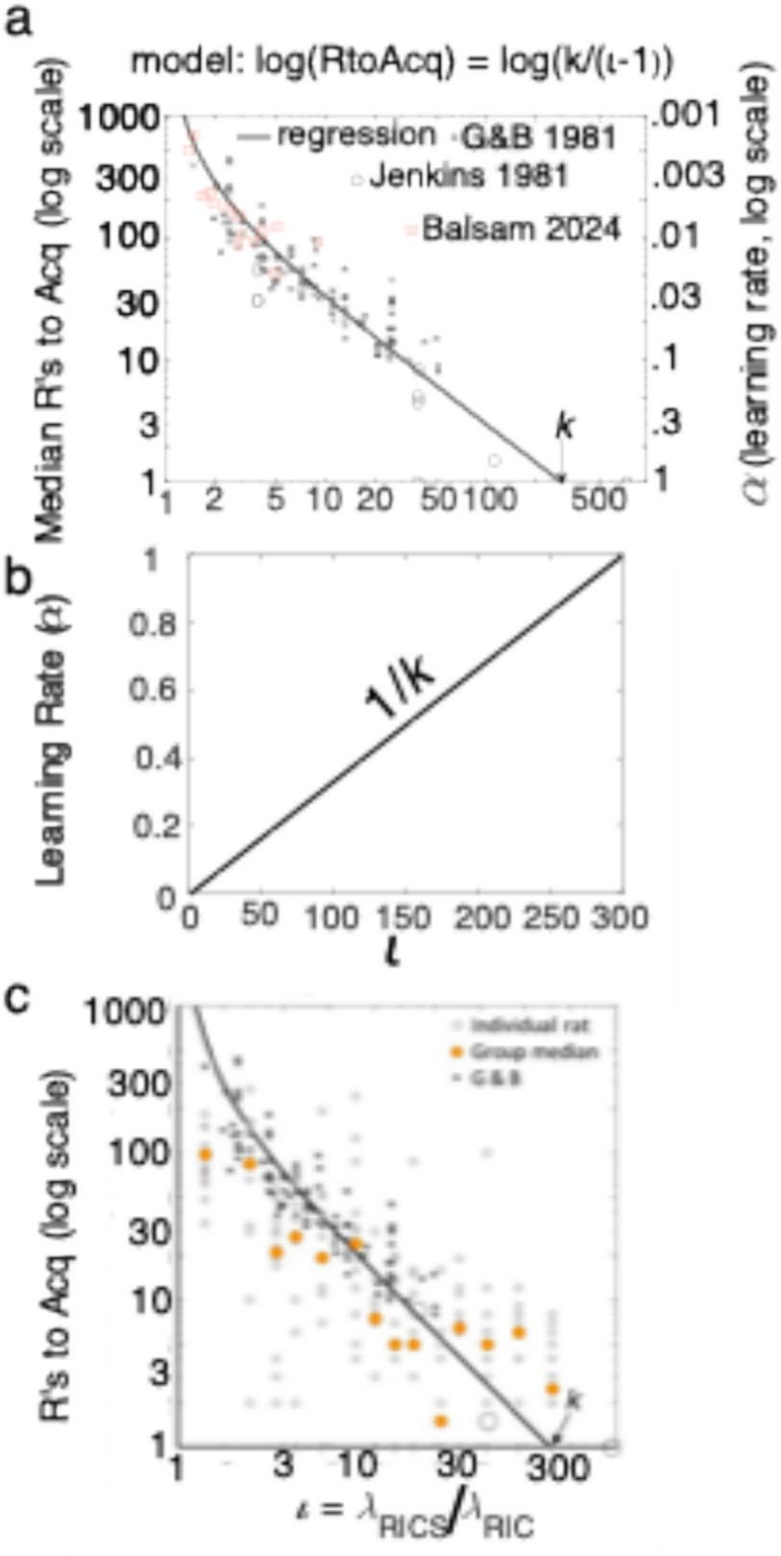
*Note.* **a.** Median reinforcements to acquisition as a function of informativeness, on double logarithmic coordinates. The regression model was fit to the asterisks only but it predicts the Jenkins out-of-sample data (open circles), which extend the empirical support to the analytic limit of acquisition after 1 reinforcement. It also predicts the results from Balsam et al. (2024) with rat subjects and reinforcements only during the ITIs (open pink squares). **b.** The regression model plotted against linear coordinates to show the almost scalar relation between the learning rate and informativeness. **c.** Data from Harris and Gallistel (2024) experiments with rat subjects and R at termination of variable duration CSs (medians are the orange circles; small open circles are individual rats). The informativeness is the x axis in all three plots

They plotted median reinforcements to acquisition as a function of informativeness (*C/T*) on double logarithmic coordinates (asterisks in Fig. 4a) and fit the data with several different regression models, the simplest of which is plotted along with their data and some of the data from the Jenkins et al. (1981) experiments (open circles). Also plotted are data from an experiment by Balsam et al. (2024) with rat subjects and reinforcements scheduled by a Poisson process that ran only during the ITIs (open pink squares). The same regression model (top of Fig. 4a) describes all three data sets despite the differences in species and many protocol details.

The regression model has only one parameter, *k*. Its value has a simple and theoretically important interpretation; it is the informativeness that produces one-trial acquisition in the median subject.

As the informativeness approaches 1 at the left end of the plot, its log, the association between the CS and reinforcement approaches 0, reinforcements to acquisition tend toward infinity, and the reciprocal of reinforcements to acquisition (the learning rate, right axis) tends to 0. The curvilinear log-log plot obscures what becomes apparent when one replots the regression model on linear coordinates as the learning rate versus informativeness. Taking antilogs on both sides and reciprocating both sides, one sees that the learning rate is a very nearly scalar function of informativeness (Fig. 4b). This maximally simple relation between the learning rate and the informativeness is the Learning Rate Law—Eq. (5).

5 $$\alpha ={~}^{1}\!\left/ \!{~}_{RtoAcq}\right.=\left(\frac{1}{k}\right)\iota -\frac{1}{k}\cong \left(\frac{1}{k}\right)\iota , 1\le \iota \le k$$

Equation (5) applies broadly, but the value for *k* is at least somewhat dependent on species and/or protocol details. Burke et al. (2023, their Fig. 1F) report reinforcements to acquisition data for trace conditioning for water reinforcement in thirsty head-fixed mice at informativeness values of 60 and 600. The median $RtoAcq$ in the $\iota =60$ group was 92 with a range from 59 to 150 for individual subjects. In the $\iota =600$ group, the median $RtoAcq$ was 8.5 with a range from 5 to 14 for the individual subjects. Given $RtoAcq$ and solving for *k,* we have $k=RtoAcq\left(\iota -1\right)$. This formula gives *k* = 5428 [–1919 +3450] for the $\iota =60$ group and k = 5092 [–2097 +3294] for the $\iota =600$ group. These calculations show first that the Law of the Learning Rate allows us to predict the data from one group given the data from another despite a tenfold difference in ITI (60s to 600s). They also show that the informativeness required for one-shot learning in a trace-conditioning protocol with head fixed mice undergoing neurobiological activity recording is about 20-fold greater than is required for ordinary conditioning in pigeon and rat. Further research can determine which factor explains the large difference in *k*: the species, the trace protocol, or the fixation of the head for the recording of neural activity. The generalization from the pigeon and rat data to these data and to rabbit eyeblink data (Gallistel & Gibbon, 2000, their Fig. 10) means that the *form* of the law is well-established. It holds across wide differences in species and protocol. It goes unmentioned in reviews that focus on the Rescorla-Wagner model and its descendants (Esber et al., 2025; Kang et al., 2024; Piray & Daw, 2021).

**Fig. 5:**
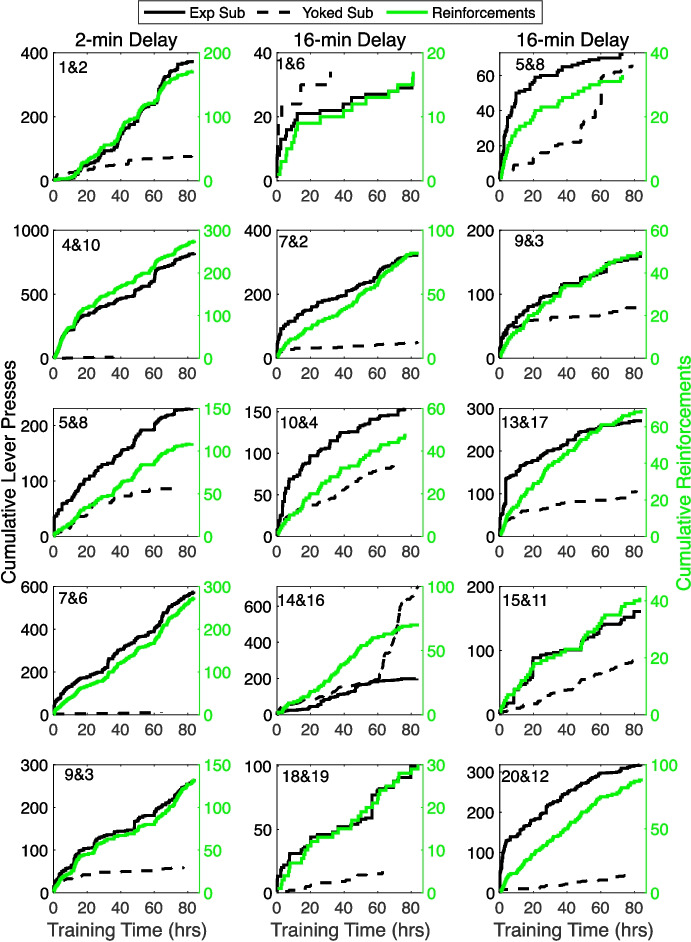
Cumulative Lever Presses versus Training Time Plotted in Black against the Left Axes; Cumulative Reinforcements versus Training Time Plotted in Green against the Right Axis. *Note. *Solid black plots are experimental subjects’ data; dashed black are yoked controls. Duration of dead delay between press and reinforcement indicated at top of columns. (A version without the plot of reinforcements appears as Fig. 1 in Gallistel & Shahan, 2024)

The law also applies to operant protocols (reinforcement learning)—with values for the one-shot learning constant, *k,* similar to those obtained from Pavlovian protocols. Inspired by the Pavlovian findings, Gallistel and Shahan (2024) found one-shot learning of an operant lever-press response in rats. They reduced the contextual rate of reinforcement and the subjects’ rate of responding by prolonged context extinction prior to the first appearance of the lever. When the lever finally appeared, a press by an experimental subject triggered reinforcement after a 2-min delay in a first experiment and after a 16-min delay in a second. Presses made during the delay had no effect. Yoked controls received reinforcements at the same times, but their presses were without effect. In most pairs, the experimental subject pressed more frequently than the yoked control after the first reinforced press (Fig. 5).

The slopes of the experimental subjects’ cumulative response records in Fig. 5—their press rates—immediately exceeded those of their yoked controls in most pairs, which indicated that the experimental subject perceived the association on its first occurrence and immediately decided to act on it. The prospective and retrospective informativeness of responses and outcomes for experimental subjects were in the range of 6 to 32. These values produce one-shot conditioning in some subjects in Pavlovian protocols (small open circles in Fig. 4c). The *n*D_KL_ measure of the strength of the evidence reached conventional levels of significance for experimental subjects within the first few reinforcement-triggering responses. In short, this model provides a quantitatively consistent model of associative learning in Pavlovian and operant (reinforcement learning) protocols.

In the Gallistel and Shahan experiments with long-delay reinforcement, an act-outcome association experienced only once produced operant responding despite a delay of reinforcement more than four orders of magnitude longer than the conventional delay (< 0.1s). Lett (1975) demonstrated learning of the correct response in a T maze with a 1-hr interval between the correct response and food reinforcement. To prevent explanation by secondary reinforcement, he removed his rat subjects from the chosen arm immediately and returned them to the stem for reinforcement or nonreinforcement after 1 hr.

In short, prospective and retrospective associative learning are time-scale invariant. The long-held belief that there is a critical window for the formation of an Aristotelean association, reiterated in most secondary sources as established fact, is simply false. When informativeness is made large enough, most subjects learn after one or two reinforced CSs or reinforced responses, no matter how long the interval between the two events. This is not learning by gradient descent (Richards & Kording, 2023).

The approximately scalar dependence of the learning rate on time-scale-invariant informativeness has far-reaching practical and theoretical consequences. As a practical matter, researchers who crowd trials together in the hope of shortening training time defeat their purpose. The duration of the training required for a conditioned response to appear is determined by the choice of the mean latency from CS onset to reinforcement. With a 5-s CS duration and a 20-min average interval between reinforced CSs (an informativeness of 250) and a session length just longer than the wait for the 2nd CS (roughly 40 min), a majority of subjects will make a conditioned response on the 2nd presentation of the CS (one-shot conditioning). Most subjects will have done so by the end of the second session (4 CS-reinforcement associations). Shortening the intertrial interval to 5 s produces an informativeness of (5 + 5)/5 = 2, which moves the regression in Fig. 4 into the region where it curves upward toward infinity (0 learning rate). Therefore, the number of reinforcements required will increase by more than a factor of 60. Training will take longer than the 40–80 min it would have taken using well-spaced trials. The reason most researchers think associative learning is slow is because they are impatient and they erroneously assume their subjects will take less time to acquire if they give them lots of trials in little time.

There is another way of putting this counterintuitive fact: suppose that for a given mean CS duration and mean intertrial interval, the median subject begins to respond after 48 reinforced trials. If one takes another group and presents them with eight times fewer trials spaced eight times more sparsely, they acquire after the same amount of training time as the first group (Burke et al., 2023; Gottlieb, 2008); that is, they acquire after eight times fewer reinforcements. When Gottlieb tried to publish this result—which he had replicated in six versions of the experiment, some within- and some between-subjects—an anonymous reviewer wrote, “Only a few crazies in Gallistel’s lab could believe that the number of reinforcements doesn’t matter.”^1^ I mention this to emphasize the extent to which most of the research community erroneously believes that many trials and short intervals between events are the key to rapid associative learning. That is true for large language models running on super computers but not for associative learning running in brains.

The approximately scalar relation between informativeness and the learning rate explains another equally counterintuitive experimental result: Partial reinforcement—interspersing reinforced trials with unreinforced trials—has no effect on the number of reinforcements required for the acquisition of the conditioned response (Gibbon et al., 1980). Partial reinforcement decreases the conditional rate, ${\lambda }_{\text{R}|\text{CS}}$, and the contextual rate, ${\lambda }_{\text{R}|\text{C}}$, by the same factor. Their ratio, the informativeness, is unchanged, and so, therefore, is the learning rate. The CS and the reinforcement are equally strongly associated; only the time scale has changed—and associative learning is time-scale invariant!

Perhaps the most counterintuitive experimental result predicted by the Learning Rate Law is that partial reinforcement in trace conditioning *reduces* reinforcements to acquisition (Burke et al., 2023). The gap between CS offset and reinforcement is the reinforcement-predicting state in trace conditioning (Balsam, 1984; Balsam & Gibbon, 1982). Put another way, reinforcement is associated with CS offset at the beginning of the gap state. On trials when no reinforcement follows, there is no gap state, so those trials do not enter the computation of the rate of reinforcement during the gaps. The informativeness of a gap is the ratio between the gap rate of reinforcement, 1/gap duration, and the contextual rate. The contextual rate is the cumulative reinforcement count divided by the cumulative duration of the intertrial intervals, whether an ITI contains a gap state or not. Partial reinforcement reduces the contextual rate without affecting the gap rate, thereby increasing the informativeness, which reduces reinforcements to acquisition.

The counterintuitive experimental results just summarized are hard to reconcile with the Aristotelean conception of associative learning. So is one-shot learning because Eq. (1) is a model for gradient descent. Some modelers in the Aristotelean tradition believe that if a process is not gradient descent, it is not a neurobiological learning process (Richards & Kording, 2023). Gradient descent in a billion-dimensional weight space is what the back-propagation algorithm in large language models does. It is slow because the changes on any given trial must be small. This perhaps explains why the Learning Rate Law, time-scale invariance and one-shot learning are rarely if ever mentioned in reviews of formal models of the Aristotelean association-forming process.

## Assignment of Credit

Rescorla and Wagner sought an explanation of the assign-of-credit results in associative learning experiments when they developed Eq. (1), the delta-rule updating equation that has dominated formal modeling (Esber et al., 2025). The assignment-of-credit problem is to determine which predictors get credit for a reinforcement. More than one predictor is generally present because CSs, responses and reinforcements occur in some context and the context competes with the CS or the response for credit. When the rate of reinforcement is the same whether the CS is present or not, the context gets credit for the reinforcements that occur during the CS (background conditioning; Rescorla, 1968). If a new CS is presented in compound with an already conditioned CS, the new CS gets none of the credit (blocking Kamin, 1969). If two CSs are always presented together, one of them gets all of the credit (overshadowing: Kamin, 1969; Reynolds, 1961). When one CS accounts for all the explainable variance, it gets all the credit even though it is reinforced no more frequently than the other two (relative validity: Wagner et al., 1968). Subjects seem to have a statistician in their brain telling them which CSs best predict. Rescorla and Wagner were trying to describe formally the brain process that enables this display of intelligence.

Equation (1) has two free parameters, the learning rate, $\alpha$, which is a scalar and the asymptotic association, $\Lambda (R)$. The latter is a vector-valued function of *R*; it has a positive value (typically 1) on reinforced trials and value 0 on unreinforced trials. In descendants of Eq. (1), there are many more free parameters (Honey et al., 2020; Piray & Daw, 2021; Vogel et al., 2019). They are adjusted during simulations to produce results resembling those obtained experimentally. They are rarely estimated experimentally, unlike *k* in the Learning Rate Law. The indefensible assumption that made Rescorla and Wagner’s simulation of Rescorla’s contingency-not-pairing results appear to work, which has remained almost unchallenged for decades, should raise suspicions about contemporary simulations that take advantage of even more free parameters.

There are no difference equations and no free parameters in the rate-based information-theoretic approach to associative learning. Because independent rates are additive, assignment of credit reduces to solving the relevant system of simultaneous equations. The observed rates of reinforcement are the known (directly observed) quantities on the right in these equations. The rates to be attributed to the possible predictors appear on the left under hats and multiplied by hatted coefficients. The coefficients are temporal probabilities, the ratios of two cumulative durations. For example, $\frac{{T}_{\text{CS}}}{{T}_{\text{C}}}$, which is the cumulative duration of the CS divided by the cumulative duration of the context, is the probability that reinforcements randomly distributed in the context will fall in a CS. The system of equations is solvable because the sum of the rates attributed to the different possible predictors when multiplied by the corresponding temporal probabilities must equal the observed rates.

The rate observed during the CSs is ${{\lambda }_{\text{R}|\text{CS}}=n}_{\text{R}|\text{CS}}/{T}_{\text{CS}}$, where ${n}_{\text{R}|\text{CS}}$ is the cumulative count of reinforcements during the CSs and ${T}_{\text{CS}}$ is cumulative CS duration (where cumulation extends across sessions). The rate observed in the context is ${{\lambda }_{\text{R}|\text{C}}=n}_{\text{R}|\text{C}}/{T}_{\text{C}}$, where ${n}_{\text{R}|\text{C}}$ is the sum of the reinforcements during CSs and the reinforcements in their absence (during the intertrial intervals). The system of simultaneous equations is

$$\frac{{T}_{\text{C}}}{{T}_{\text{C}}}{\widehat{\lambda }}_{\text{C}}+\frac{{T}_{\text{CS}}}{{T}_{\text{C}}}{\widehat{\lambda }}_{\text{CS}}={\lambda }_{\text{R}|\text{C}}$$

and

$$\frac{{T}_{\text{C}|\text{CS}}}{{T}_{\text{CS}}}{\widehat{\lambda }}_{\text{C}}+\frac{{T}_{\text{CS}}}{{T}_{\text{CS}}}{\widehat{\lambda }}_{\text{CS}}={\lambda }_{\text{R}|\text{CS}}.$$

The first equation says that the contextual rate on the right, ${\lambda }_{\text{R}|\text{C}}$, equals the rate to be attributed to the context itself, ${\widehat{\lambda }}_{\text{C}}$, plus the fraction of the time the CS was present, $\frac{{T}_{\text{CS}}}{{T}_{\text{C}}}$, times the rate to be attributed to the CS, ${\widehat{\lambda }}_{\text{CS}}$. The second says that the observed CS rate on the right, ${\lambda }_{\text{R}|\text{CS}}$, is the sum of the rate attributed to it and the rate attributed to the context, ${\widehat{\lambda }}_{\text{C}}$, because, when the CS is present, so is the context.

In matrix form, the system of equations is written

6a $${\widehat{{\varvec{\lambda}}}}_{\text{R}}={\left[\begin{array}{cc}\frac{{T}_{\text{C}}}{{T}_{\text{C}}}& \frac{{T}_{\text{CS}}}{{T}_{\text{C}}}\\ \frac{{T}_{\text{C}|\text{CS}}}{{T}_{\text{CS}}}& \frac{{T}_{\text{CS}}}{{T}_{\text{CS}}}\end{array}\right]}^{-1}\left[\begin{array}{c}{\lambda }_{\text{R}|\text{C}}\\ {\lambda }_{\text{R}|\text{CS}}\end{array}\right]={\left[\begin{array}{cc}1& p(\text{CS}|\text{C})\\ 1& 1\end{array}\right]}^{-1}\left[\begin{array}{c}{\lambda }_{\text{R}|\text{C}}\\ {\lambda }_{\text{R}|\text{CS}}\end{array}\right]$$

In the conventional notation for a matrix equation, this becomes wonderfully compact:

6b $${\widehat{{\varvec{\lambda}}}}_{\text{R}}={\mathbf{P}}^{-1}{{\varvec{\lambda}}}_{\text{R}}$$

where ${\widehat{{\varvec{\lambda}}}}_{\text{R}}$ denotes the column vector of *attributed* rates (the unknowns), ${\mathbf{P}}^{-1}$ denotes the inverse of the conditional probability matrix, and ${{\varvec{\lambda}}}_{\text{R}}$ is the column vector of *observed* rates of reinforcement (the knowns).

The elements of the matrix are the coefficients of the equations. The coefficients are the conditional temporal probabilities of the potential predictors. Unlike the most common probabilities, which are ratios of unitless counts, temporal probabilities are ratios of cumulative durations. The temporal units cancel, leaving unitless, time-scale-invariant temporal probabilities. The temporal probabilities are observed, hence measured. The model assumes hatted variables in the head approximately equal to the observed probabilities.

The upper left coefficient, $\frac{{T}_{\text{C}}}{{T}_{\text{C}}}$, in the matrix in Eq. (6a) is the probability that the context is present conditional on its being present, which is, of course, 1. The upper right coefficient, $\frac{{T}_{\text{CS}}}{{T}_{\text{C}}}$, is the probability of the CS being present at randomly chosen moments when in the context. When there are no reinforcements other than during the CS, this ratio is the reciprocal of the informativeness. The lower left coefficient, $\frac{{T}_{\text{C}|\text{CS}}}{{T}_{\text{CS}}}$, is the probability that the context is present when the CS is present; it is 1 because the CS occurs only in the test chamber. The bottom right coefficient is the probability that the CS is present given that it is present, which is, of course, 1.

The –1 exponent on the matrix denotes inversion. The matrix is inverted by the Gaussian row-echelon elimination algorithm, now built into spreadsheets and scientific programming languages. The inverted matrix is a mathematical machine; it takes observed rate vectors as input and outputs attributed rate vectors. Like all mathematical machines, its gears are the basic binary operations in arithmetic (± and $\times$/÷).

For illustration, consider the application of Eq. (6a) to Rescorla’s contingency-not-pairing experiment, in which he employed the truly random control (Rescorla, 1968)—see Fig. 1: an “experimental” group was shocked at a rate of approximately 0.25/min only during 2 min-long tone CSs that were separated on average by roughly 18 min. The “truly random control” group—the group of principal interest—was shocked at the same rate throughout each session, whether the CS was present or not. The rats in the experimental group learned to fear the CS; the rats in the “control” group did not—although they got the same temporal pairings between CSs and shocks as the experimental group got. They did, however, learn to fear the chamber. They resisted being placed into it.

For both groups, the CS was present 1/10th of the time, so for the matrix we have:

$$\mathbf{P}=\left[\begin{array}{cc}1& .1\\ 1& 1\end{array}\right].$$

Notice that the only element of the matrix not equal to 1 is the reciprocal of the informativeness. In the new conception of associative learning, informativeness appears almost everywhere. It ties different problems and results together conceptually. For the experimental group, the contextual rate of reinforcement was 1/10th of the CS rate, so we have:

$${\widehat{{\varvec{\lambda}}}}_{\text{R}}={\left[\begin{array}{cc}1& .1\\ 1& 1\end{array}\right]}^{-1}\left[\begin{array}{c}.025\\ .25\end{array}\right]=\left[\begin{array}{c}0\\ .25\end{array}\right].$$

Credit for the shocks is attributed entirely to the tone CS. For the truly random control the observed rates are both 0.25, so we have:

$${\widehat{{\varvec{\lambda}}}}_{\text{R}}={\left[\begin{array}{cc}1& .1\\ 1& 1\end{array}\right]}^{-1}\left[\begin{array}{c}.25\\ .25\end{array}\right]=\left[\begin{array}{c}.25\\ 0\end{array}\right].$$

Credit for the shocks is attributed entirely to the test chamber (the context). Anyone with access to a spreadsheet can verify these computations; no simulation is required.

The rate of reinforcement *attributed* to the context in the experimental group is not the same as the *contextual* rate of reinforcement; the attributed rate is 0, whereas the contextual rate is 0.025. The nonzero contextual rate of reinforcement explains why the rats in the experimental group resisted being put into the test chamber even though they attributed 0 rate of shock to it. They feared the chamber because it predicted the CSs during which they sometimes got shocked.

This model of credit assignment distinguishes between what can be expected in a context—the contextual rate of reinforcement—and the rate attributed to the context itself. The distinction inheres in the mathematics, because the contextual rate is one of the knowns in the system of simultaneous equations. If a subject does not estimate it, the subject cannot solve the assignment of credit problem. Its inclusion in the model enables this model of credit-assignment to explain the black data points in Fig. 1; the rats’ response rate during the ITIs depends on the contextual rate of reinforcement, not on the rate attributed to the context. The simple mathematics in the new conception connect seemingly unrelated results. They integrate concepts and results.

Equation (6b) explains all of the results that Eq. (1) is claimed to explain (Gallistel, 1990, ch. 13)—and some results that it is acknowledged not to explain, notably retroactive blocking and unblocking (Blaisdell et al., 1999; Matzel et al., 1985). Equation (1) does not explain Rescorla’s (1968) results; nor do subsequent delta-rule updating models inspired by it. His scheduling of reinforcements with a Poisson process made it impossible to specify when nonreinforcements occurred and, a fortiori, how many there were (Gallistel, 2021b). Any model that decrements something when and at the time when a reinforcement fails to occur cannot explain those results.

Equation (6b) is explicit and parameter-free, unlike the generally unsolved difference equations in parameter-rich delta-rule updating models (Honey et al., 2020; Rescorla & Wagner, 1972; Vogel et al., 2019). There is no wiggle room. It predicts reinforcement-by-reinforcement in real time the evolution of attributions in individual subjects. It is a direct consequence of the insight that associative learning is driven primarily by the perception of relative rates of reinforcement, not by the updating effects of reinforcements and nonreinforcements on Aristotelean associations. Because attributed rates are directly manifest in measurable rates of responding (Fig. 2), we can track them in real time in individual subjects.

Equation (6b) is relevant in Pavlovian protocols where the subject’s behavior has no effect on the communicated information between CSs and reinforcements. In operant protocols, assignment-of-credit is solved by the perception of the information communicated between responses and reinforcements. The operant contingencies depend on the subject’s behavior. In both types of protocols, informativeness is the crucial variable. The log of informativeness measures the association and the *n*D_KL_ measures the strength of the evidence for it.

## Change-Detection

Estimating a rate by dividing the count by the duration over which events were counted implicitly assumes the rate was constant during the count. The same stationarity assumption is implicit when estimating a probability. In fact, however, rates and probabilities may change. The classic example in the experimental study of associative learning is extinction; when Pavlov stopped delivering food at the termination of the CS, his dogs eventually stopped salivating to it.

It has always been understood that Hullian models, including the Rescorla-Wagner model and contemporary delta-rule updating reinforcement learning models inspired by it, do not explain long-standing experimental results on extinction and recovery from it (Gleitman et al., 1954; Kang et al., 2024; Kimble, 1961; Niv & Schoenbaum, 2008). Consider the simplest example in which one delivers reinforcements on a Poisson schedule in a test chamber and then stops doing so, either mid-session or at the start of a new session. During the period when the reinforcements randomly occur, subjects run around looking for them. The rate at which they run around is proportional to the rate of reinforcement. When one stops delivering reinforcements, they eventually stop running around (Killeen, 2023; Killeen & Sitomer, 2003; Lea & Dow, 1984). One is inclined to attribute the change in their behavior to the causal effects of nonreinforcements, that is, to a failure of expectations, with each failure decrementing the association between the context and reinforcement. But when the reinforcements were randomly scheduled, this attributes causal efficacy to immaterial ~R’s, “events” that have no sensory effects and whose times of occurrence cannot be specified. When that is true, the ~R’s cannot be counted and a ~R cannot cause the decrementing of net associative strength. Once again, the problem is that Eq. (1) takes no account of the durations of the intervals between events.

The most challenging experimental result is the partial reinforcement extinction effect (Kimble, 1961, p. 286ff). If ~R’s decrement net associative strength, intermingling unreinforced CS presentations with reinforced presentations should weaken the association at the end of training. It should then require fewer consecutive unreinforced CSs to produce extinction. For decades, the opposite has been known to be true. Partial reinforcement increases the number of consecutive unreinforced trials required to meet some criterion of extinction. The effect is scalar. When only 1 in 10 training trials is reinforced, it takes 10 times as many consecutive unreinforced CS presentations to produce extinction (Gibbon et al., 1980).

Subjects responding on concurrent variable interval schedules in which the relative rates of reinforcement change frequently approximate ideal detectors of the changes in the relative rates. They adjust to them abruptly and about as quickly as if they were getting advice from a statistician (Gallistel et al., 2001). This implies that brains deal with nonstationarity by applying a good real-time parsing algorithm to the data in the temporal map. The algorithm detects changes and reports an estimate of when in the past they occurred. This report makes it possible to truncate the data on which the current estimate of a rate or probability is based at the point just after the most recent change.

From this perspective, changes are themselves events and are recorded as such on the temporal map, thereby enabling the brain to detect simple patterns in the changes (Higa et al., 1991; Ricci & Gallistel, 2017). Truncating the data on which the current estimate of a rate or probability is based prevents it from becoming an average over epochs in which there were two very different rates, neither of which would be correctly represented by the current estimate if it ranged over data from both epochs. Because they do not parse a temporal map, models inspired by Eq. (1) average across changes. They do not, therefore, correctly predict extinction and other examples of the behavioral changes that occur in response to changes in the parameters of stochastic processes (Lea & Dow, 1984).

The key to effective change-detection is to realize that the point slope of a cumulative record of events is the event rate at that point in time. A cumulative record of reinforcements is a plot of the count as a function of time (or, for probability change detection, as a function of trials). A change in the event rate creates a noticeable elbow in the cumulative record (Gallistel et al., 2004; see the cumulative records in Fig. 5.)

A simple real-time elbow-detecting algorithm uses Eq. (4), the formula for the *n*D_KL_, the statistic that measures the reliability of an association. I have already assumed that the brain computes it reinforcement by reinforcement to judge the reliability of perceived associations. As long as the rate has not changed, the *n*D_K_ values bounce around beneath the gamma(.5,1) probability density distribution (see Peter Latham's Appendix to Gallistel & Latham, 2022). When the estimates of a rate of reinforcement span a change, the current estimate will no longer be consistent with earlier estimates. If the change was to a lower rate (a downward elbow in the cumulative record), the current estimate will be bigger than the actual current rate and smaller than the pre-change rate—and vice versa if the change was to a higher rate (an upward elbow).

The change-detecting algorithm uses Eq. (4) to compare the sequence of previous rate estimates (in the numerator of the informativeness ratio) to the current rate estimate (in the denominator). The *n*D_KL_‘s get steadily larger up to the point in the temporal map where the change occurred; then they decline all the way to the current time. The location of the maximum in the *n*D_KL_ sequence estimates the point in the past where the change occurred; it coincides with the elbow. The brain sets a decision criterion on the maximum. When the maximum exceeds the criterion, the algorithm reports a change and where it occurred. Thereafter, the data on which the current rate estimate is based go back only to just after that most recent change point.

Because the algorithm operates on an *n*D_KL_ vector the formula for modelling change detection is:

7 $$i={\text{i}}_{\text{max}}({\varvec{n}}{\mathbf{D}}_{\text{KL}},k)\left[\begin{array}{l}\text{max}\left({\varvec{n}}{\mathbf{D}}_{\text{KL}}\right)>k\\ 0 \text{ otherwise}\end{array}\right]$$

The D_KL_ function embedded in the ${\text{i}}_{\text{max}}$ function is distribution-specific; the D_KL_ in Eq. (7) has one form for changes in rate and a different form for changes in probability. Let ${\varvec{t}}=[{t}_{0}, {t}_{1},\dots {,t}_{i},\dots {,t}_{n},t$] be the time-stamp vector for reinforcements. Its elements are ${t}_{0}$, the time at which the count of reinforcements began, ${t}_{i}$, the time of each counted reinforcement, and $t$, the current time. Let ${\varvec{i}}=[\text{1,2},\dots ,n]$ be the integer indices for the elements of ${\varvec{t}}$. Then

$${\varvec{\lambda}}=\frac{{\varvec{i}}}{{\varvec{t}}\left[1:n\right]}$$

is a reinforcement-by-reinforcement vector of reinforcement rate estimates;

$$\lambda \left(t\right)=\frac{n}{t-{t}_{0}}$$

is the rate estimate as of the current time $t$; and

$${{\varvec{n}}}_{{\varvec{e}}}={\varvec{i}}./\left(1+\frac{{\varvec{i}}}{n}\right)$$

is the vector of effective sample sizes.

To compute a change point for a *probability,* the brain does not count reinforcements that failed to occur. The probability estimation process counts events strongly associated with reinforcements, for example, the CS offsets in a Pavlovian delay-conditioning. In those protocols, every reinforcement coincides with a CS offset (whereas the reverse is not true if CSs are only partially reinforced). The *retrospective* contingency is 1, because for every reinforcement there is a CS offset with the same time stamp. In that case, ${\varvec{t}}$ is the vector of CS-offset times, not reinforcement times. Associated with it is the equinumerous vector ${{\varvec{n}}}_{\text{R}}$, which gives the count of the reinforcements that have coincided with the trial offsets. Then

$${\varvec{p}}=\frac{{{\varvec{n}}}_{\text{R}}}{{\varvec{i}}}$$

is the vector of estimates for ${p}_{\text{R}}(i)$, the probability of reinforcement, as of the *i*^th^ trial, and

$$p\left(n\right)$$

is the current estimate. When estimating rate changes, we then have:

7a $${\varvec{n}}{\mathbf{D}}_{\text{KL}}\left({\varvec{i}}\right)={{\varvec{n}}}_{{\varvec{e}}}\left[\text{ln}\frac{{\varvec{\lambda}}}{\lambda \left(t\right)}+\frac{\lambda \left(t\right)}{{\varvec{\lambda}}}-1\right],$$

and when estimating probability changes, we have:

7b $${\varvec{n}}{\mathbf{D}}_{\text{KL}}\left({\varvec{i}}\right)={{\varvec{n}}}_{{\varvec{e}}}\left[{\varvec{p}}\text{ln}\frac{{\varvec{p}}}{p\left(n\right)}+\left(1-{\varvec{p}}\right)\text{ln}\frac{1-{\varvec{p}}}{1-p(n)}\right]$$

When there has been no change in the rate, the elements of ${\varvec{n}}{\mathbf{D}}_{\text{KL}}$ in Eq. (7) are distributed gamma(.5,1) (Gallistel & Latham, 2022). The index returned by the ${\text{i}}_{\text{max}}$ function when it detects a change estimates the reinforcement, hence the ${t}_{i}$, at which the change occurred. The only parameter in this model of change detection is the decision criterion. When Eq. (7) is applied to extinction data, it estimates the decision criterion. Thereafter, the decision criterion is no longer a free parameter.

Although rate of reinforcement plays the central role in the reconceptualization of associative learning, subjects do estimate probability of reinforcement when it is defined. Estimated probability of reinforcement plays a role in determining various aspects of the resulting behavior (Mallea et al., 2024). For example, the change in the probability of reinforcement is the key variable in the extinction of Pavlovian delay conditioning (Bouton et al., 2014; Harris & Andrew, 2017) because reinforcements, when they occur, coincide with CS terminations, so the retrospective contingency is 1.

**Fig. 6:**
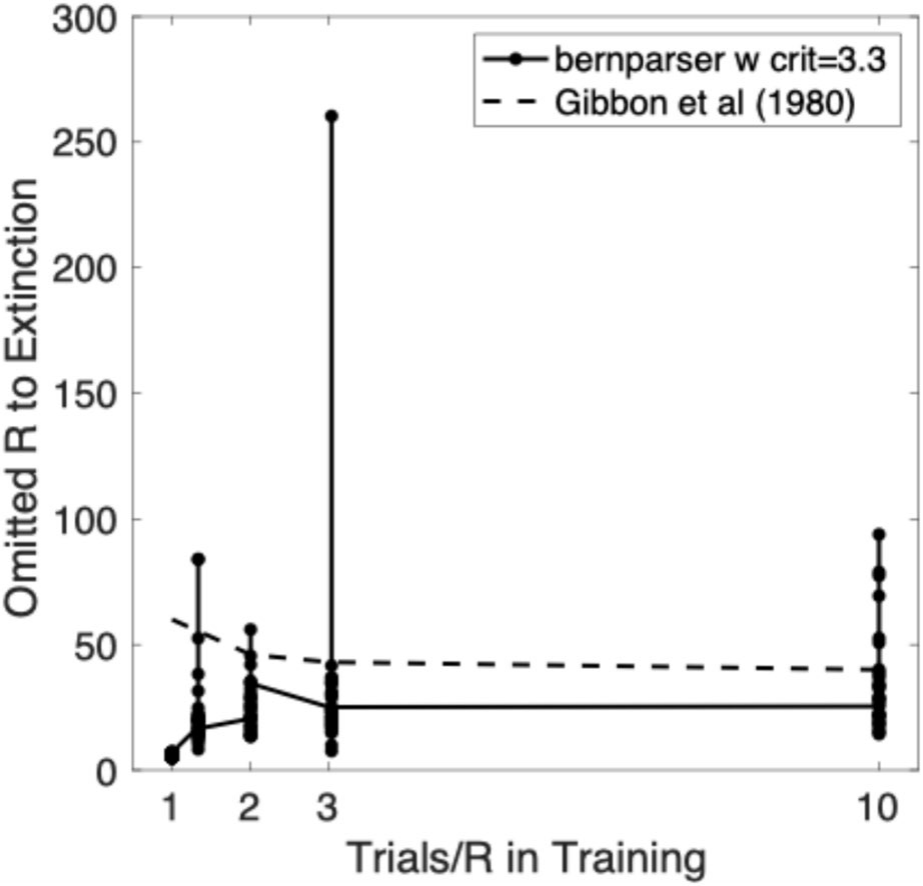
Omitted Reinforcements to Extinction Plotted against Trials per Reinforcement during Training. *Note.* Dashed plot derived by applying a level curve to Gibbon et al. (1980, Fig. 4, p. 49). Dots connected by a solid line are the omitted reinforcements to extinction predicted by applying Eq. (8b) to simulated sequences of binary training data at the 4 different values for probability of reinforcement (.75, .5, .33, and .1) followed by a long extinction sequence of nonreinforcements (‘0’s). There were 16 simulations at each of the 4 probabilities of reinforcement. The assumed decision criterion was 3.3 nats, which corresponds to odds of 100:1 against the null (no-change) hypothesis

Equation (7b) predicts the scalar effect of partial reinforcement on trials to extinction in pigeon autoshaping (Fig. 6), a challenge that has long defeated formal models of associative learning.

In this model, changes are themselves events, stored in the temporal map. Extinction is the process of learning that what was once true is no longer true. In other words, that was then, this is now. The passage of time and any event that indicates further change induces the temporary return of behavior—on the assumption that perhaps what once was may have returned to currency (spontaneous recovery, renewal, reinstatement).

## Departure-Rate Equation

In a concurrent VI protocol, the longer the subject dwells at Location I, the more certain it is that a switch to Location J will produce immediate reinforcement. If choosing the other location is conceptualized as a choice strengthened in proportion to the immediacy of its reinforcement, and if the elapsing duration of a stay at a location is taken to be a discriminative stimulus, then the tendency to switch to the other location should increase with the prolongation of a stay. In fact, however, it does not; dwell times (visit durations) are exponentially distributed (Gallistel et al., 2001; Gibbon, 1995; Heyman, 1979; Mark & Gallistel, 1994); the hazard function is flat; the momentary probability of departing for the other location does not change as the duration of a stay gets longer. This discovery led Heyman (1982) to suggest that time allocation when foraging among different locations was not conditioned behavior; it was a product of an innate policy.

Subsequent experiments by Belke and Gibbon revealed the policy. The first element of the policy is to schedule departures with a Poisson process to make visit durations exponentially distributed. Visiting different locations is motivated as much if not more by information gathering than by food gathering. By visiting locations, subjects learn the distribution of interreinforcement intervals at each location. If there is a periodicity in their visit schedule, there is risk of aliasing. Aliasing occurs when the sampling period is similar to a period in the distribution of interreinforcement at the sample location, as there is, for example, when there is a fixed-time schedule of reinforcements. Aliasing makes the wagon wheels appear to turn backwards in the movies when the stage pulls into town and slows down. The backward turning illusion occurs when the frame rate in the camera is close to the wheel rotation rate. Exponentially distributed visit durations whiten the data, thereby preventing aliasing.

The second element in the policy is to scale the rate constants of the Poisson processes by the contextual rate of reinforcement. This adjusts the rate of running around to the overall frequency of reinforcement in a context. When reinforcements are scarce, it makes no sense to run around rapidly; when times are good, it makes no sense not to.

The third element in the innate policy is to proportion dwell durations to expected reinforcement rates (the Matching Law; Herrnstein, 1961). This equates the returns at the different locations, the amount of reinforcement per unit time invested in visiting them. It also comes close to maximizing overall return (Heyman & Luce, 1979). It is an evolutionarily stable strategy because no policy adopted by competitors can do better than this policy (Charnov, 1976).

The elements of an efficient information-gathering policy were drawn together by Gibbon in his Markov-process model (Gibbon, 1995, his Fig. 2), which yields Eq. (8), the Dwell Time Equation:

8 $$\frac{1}{{\lambda }_{\text{D}|\text{I}}}={\mu }_{\text{D}|\text{I}}=\frac{1}{{\lambda }_{\text{C}}\frac{{\lambda }_{\text{R}|\text{J}}}{{\lambda }_{\text{R}|\text{I}}+{\lambda }_{\text{R}|\text{J}}}}=\frac{1}{k\left({\lambda }_{\text{R}|\text{I}}+{\lambda }_{\text{R}|\text{J}}\right)\frac{{\lambda }_{\text{R}|\text{J}}}{{\lambda }_{\text{R}|\text{I}}+{\lambda }_{\text{R}|\text{J}}}}=\frac{1}{k{\lambda }_{\text{R}|\text{J}}}$$

where I and J denote locations or mutually exclusive states between which subjects are free to switch back and forth;

$${{\lambda}_{\text{D}|\text{I}}}$$
denotes the departure rate at one of them (departure count/cumulative dwell time);
$${\mu}_{\text{D}|\text{I}}$$
denotes its reciprocal, the mean dwell time;
$${\lambda}_{\text{R}|\text{I}}$$ and $${\lambda}_{\text{R}|\text{J}}$$
are the reinforcement rates (≈VI parameters);
$${\lambda}_{\text{C}}$$
is the cycling rate (cycles/time);
$${\text{k}}$$
is the constant of proportionality between the contextual rate of reinforcement and the cycling rate:

$${\lambda }_{\text{C}}=k\left({\lambda }_{\text{R}|\text{I}}+{\lambda }_{\text{R}|\text{J}}\right);$$

and $\frac{{\lambda }_{\text{R}|\text{J}}}{{\lambda }_{\text{R}|\text{I}}+{\lambda }_{\text{R}|\text{J}}}$ is the reinforcement rate at J relative to the contextual reinforcement rate.

**Fig. 7:**
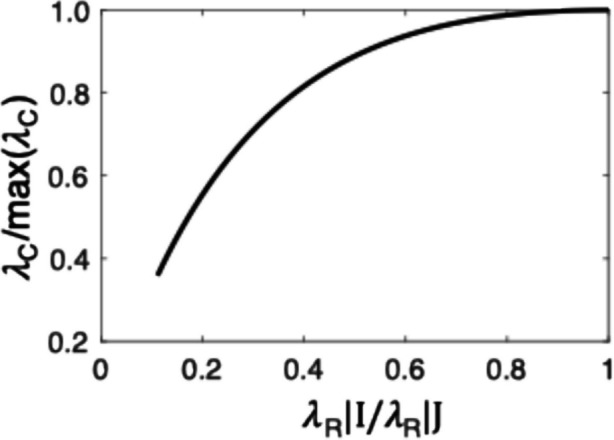
Normalized cycling rate as calculated from Eq. (8b), plotted against the ratio of the lower rate of reinforcement, ${\uplambda }_{\text{R}}|\text{I}$, to the higher, ${\uplambda }_{\text{R}}|\text{J}$. *Note. *See Alsop and Elliffe’s (1988, their Fig 4, p. 28) for plots of comparable experimental results

Herrnstein’s matching Law follows by simple substitution:

8a $$\frac{{\mu }_{\text{D}|\text{I}}}{{\mu }_{\text{D}|\text{J}}}=\frac{\frac{1}{k{\lambda }_{\text{R}|\text{J}}}}{\frac{1}{k{\lambda }_{\text{R}|\text{I}}}}=\frac{{\lambda }_{\text{R}|\text{I}}}{{\lambda }_{\text{R}|\text{J}}},$$

as does the equation for the cycling rate as a function of the two reinforcement rates (see Fig. 7 for plot):

8b $${\lambda }_{\text{C}}=\frac{1}{{\mu }_{\text{D}|1}+{\mu }_{\text{D}|2}}=\frac{1}{\frac{1}{{\lambda }_{\text{R}|2}}+\frac{1}{{\lambda }_{\text{R}|1}}}$$

**Fig. 8:**
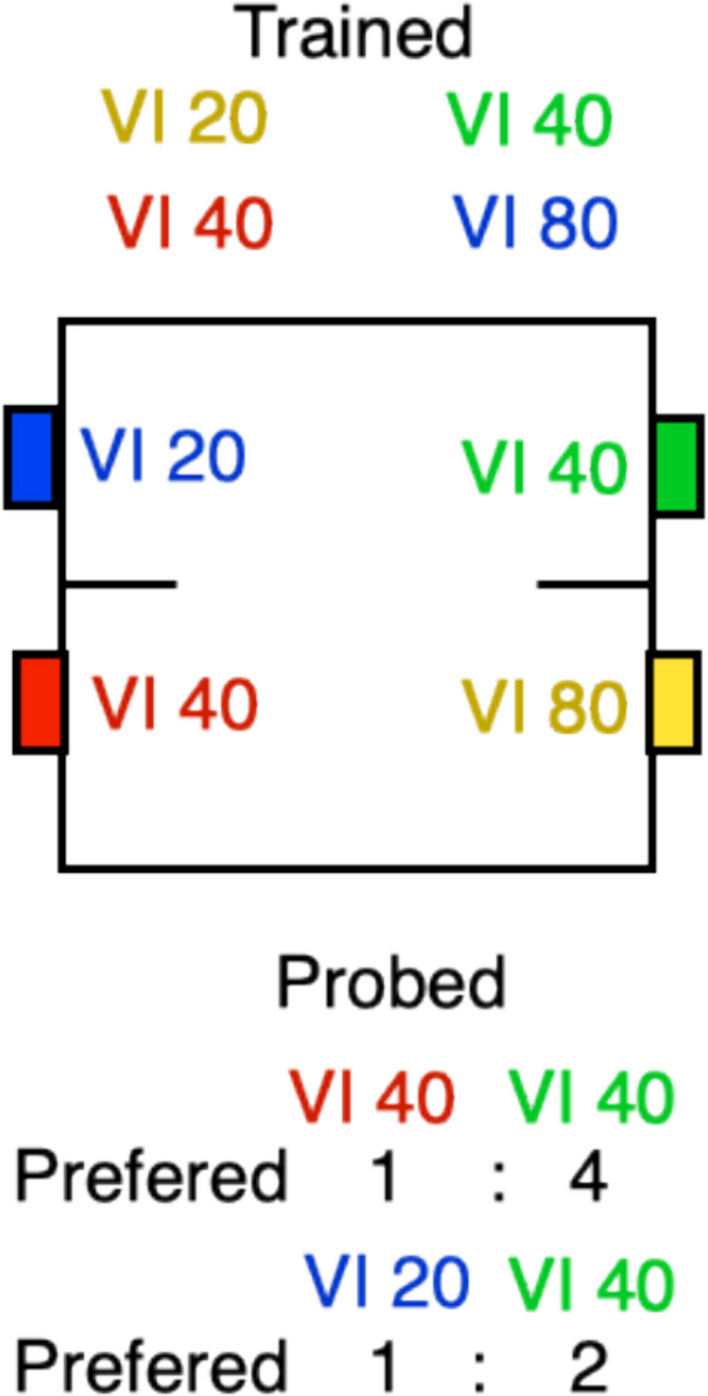
The Belke (1992) and Gibbon (1995) Revealed-Preference Experiments. (The colors in Fig. 8 are those used by Belke and by Gibbon, except that blue in Fig. 8 replaces their white. They used a change-over key protocol. Unlike in this illustration, the alternative key was not visible when a subject was pecking at a given key color.)

Equation (8) explains the counterintuitive results obtained first by Belke (1992) and again by Gibbon (1995). The Belke-Gibbon experiments measured what economists call revealed preference. They trained pigeons on two concurrent VI protocols. In each protocol, the pigeons switched back and forth between two “locations:”^2^ between the blue and red in one protocol (left side of Fig. 8) and between the green and yellow in the other (right side of Fig. 8). A VI 40s schedule was common to both protocols. It was associated with the red in one concurrent pair and with the green in the other. The alternative to VI40s in the red-blue pair was two-fold richer (VI20s); in the green-yellow pair, it was two-fold poorer (VI80s).

Belke tested well-trained birds for their revealed preference between the red and green locations, which were associated with the same VI 40s reinforcement rate but in different pairs, hence different contexts. He did this by presenting them as the only “choices” on short probe sessions with no reinforcements. The pigeons “preferred” the green 4:1; they dwelt there four times longer and made four times as many pecks. Gibbon (1995) replicated this finding. He extended it, by also testing the birds for their “preference” when the choice was between the blue (VI 20s) and green (VI 40s). They “preferred” green 2:1, even though it was associated with a rate of reinforcement only half as good as the alternative.

There are scare quotes around “preferred” and “preference,” because, on the model in Eq. (8), the pigeons were not choosing between the alternatives offered. At each location during the revealed preference tests, they ran the same Poisson process they ran at that location during training. When it timed out, they left. They then went to the other active location. There, too, the average duration of their stay remained the same as during training.

Equation (8) explains their results with quantitative rigor. Solving it for the dwell times, we have:

$$\begin{array}{l}{\mu }_{\text{D}}|\text{red}=\frac{1}{k{\lambda }_{\text{R}}|\text{blue}}=\frac{1}{k}20s\\ {\mu }_{\text{D}}|\text{green}=\frac{1}{k{\lambda }_{\text{R}}|\text{yellow}}=\frac{1}{k}80s\\ {\mu }_{\text{D}}|\text{blue}=\frac{1}{k{\lambda }_{\text{R}}|\text{red}}=\frac{1}{k}40s\end{array}$$

Ignoring *k*, which is the same in all three cases, we see that the dwell time associated with green is four times longer than that associated with red and two times longer than that associated with blue. The longer dwell times are a consequence of the longer cycling time in the poorer pair of VIs. The alternative to the VI40s rate in the richer pair was a VI 20s, whereas for the poorer, it was a VI 80s. The resulting large difference in the contextual rates of reinforcement produced faster cycling in the rich-pair context and slower cycling in the poor-pair context. Faster and slower cycling translate into shorter and longer dwell times, which is why, to quote Gibbon’s title, “Arousal makes better seem worse.” The “seem” in this quote refers to *our* perceptions, not those of the pigeons. We compare the rates of reinforcement at the two locations, between which we suppose them to have been choosing. The experimental results show that they were not comparing nor choosing; they were leaving when the Poisson process that schedules departures timed out. Here, too, it is relative rates—in this case, the departure rates—that enables us to understand the underlying process, not choice probabilities.

## Conclusions

Reconceptualizing associative learning as the perception of measurable temporal associations yields a mathematically coherent quantitative understanding of a wide range of experimental results, many of which have resisted quantitative explanation for decades. The key to the reconceptualization is the realization that for a brain to perceive a temporal association it must compute the informativeness ratio between two observed rates, a conditional rate, and the relevant contextual rate. This shifts the focus onto rate of reinforcement and rate of responding rather than probability of reinforcement and probability of response.

The shift in focus brings with it a major methodological benefit, because, under many circumstances, there is a scalar mapping from measured rates of reinforcement to measured rates of responding (Fig. 2). When this is true, we measure the brain’s representation of a quantitative fact about its experience (rate of reinforcement) when we measure rate of responding. For research on the neurobiological bases of associative learning, this is a gift.

The ubiquitous role of informativeness brings to the study of associative learning the power and elegance of information theory because the log of informativeness is the information communicated between temporally associated events—Eq. (3). Informativeness is also the variable in the formula for measuring the strength of the evidence for temporal association—Eq. (4). It is also the variable in the change-detection equation—Eq. (7)—which gives us a rigorous quantitative explanation of the partial-reinforcement extinction effect. Informativeness determines the learning rate—Eq. (5). The reciprocal of informativeness, p(CS) = $\frac{{T}_{\text{CS}}}{{T}_{\text{C}}}$, is the key element of the $\mathbf{P}$ matrix in credit assignment—Eq. (6a).

Equation (5), the Learning Rate Law, may be rewritten as a trade-off function:

$${n}_{\text{R}}\left(\iota -1\right)=k$$

In this form, the equation says that the product of the number of reinforced CSs, ${n}_{\text{R}}$, and CS informativeness $\left(\iota -1\right)$ must exceed $k$ for a subject to decide to increase its rate of responding when being trained on a simple Pavlovian protocol in which the CS predicts reinforcement. The equation specifies combinations of measured stimulus variables—${n}_{\text{R}}$ and $\iota$—that have a constant behavioral effect—the onset conditioned responding. The empirical function for omitted reinforcements to extinction as a function of trials/reinforcement during training—the dashed plot in Fig. 8—is also a trade-off function. The stimulus variables are the ~R/trial during training and the number of consecutive ~R trials during extinction.

Trade-off functions have singular importance in behavioral neuroscience, because the behaviorally obtained function must obtain for every neurobiological variable in the causal path from the point in the brain where the effects of the two stimulus variables combine to observable behavior (Gallistel et al., 1981). For example, the scotopic spectral sensitivity function obtained from the verbal responses of human subjects superimposes on the in situ spectral sensitivity of rhodopsin, because the signal detected by the human observer is jointly determined by the light intensity, its wavelength and the absorption spectrum of rhodopsin. A simple trade-off experiment makes the absorption spectrum of the key molecule at the beginning of the causal cascade evident in the behavioral data. Satisfying a behavioral trade-off function is a powerful test for a linkage hypothesis, for example, the hypothesis that the isomerization of rhodopsin is the first stage in scotopic vision. A linkage hypothesis identifies a behaviorally defined variable (e.g., scotopic spectral sensitivity) with a variable defined by physical chemistry (the absorption spectrum of rhodopsin; see Teller & Pugh, 1983, for further discussion of linkage hypotheses).

Suppose, for example, one were to conjecture that the mesolimbic dopamine signal encodes the decision variable for adjusting response rate to the change in reinforcement rate predicted by a CS. On that linkage hypothesis, the neurobiological effect of informativeness and the effect of the number of reinforcements must combine to produce a signal whose value equals a constant when conditioned behavior appears. One reinforcement with high informativeness must produce the same signal as several hundred with low informativeness. If this is not the case, then the linkage hypothesis fails. None of the linkage hypotheses proposed for the mesolimbic dopamine signal has been subject to such a strong test (see Namboodiri, 2024, for review).

The reconceptualization of associative learning changes the ontological status of “association” in the cognitive and neurosciences. Associations are no longer hypothetical mental entities often linked to postulated changes in interneuronal connections in brains (Brown et al., 1990). Associations are measurable statistical facts about the distribution of events in time. They exist independently of minds and brains.^3^

The percept of a temporal association is no more itself an association than a color percept is a surface reflectance spectrum. The percepts on which intelligent behavior in associative learning depends are symbols in memory. They represent perceived quantitative facts about the environment in which behavior unfolds, such as the conditional and unconditional rates of reinforcement. Quantity-specifying symbols are the information-carrying physical stuff on which a brain’s computational machinery operates to generate behavior (Gallistel, 1990, 2021a, b; Gallistel & King, 2010).

The change in the ontological status of “association” within the neurobehavioral study of associative learning has broad implications. It undermines the conceptual foundations for current efforts to determine the neurobiological basis of memory. Most such efforts use Pavlovian protocols to instill engrams (Poo et al., 2016; Schultz, 2015). They take for granted the motto of the Society for Neuroscience—“Neurons that fire together, wire together” (Shatz, 2019). They look in brains for the neurobiological realization of the associative bonds first posited by Aristotle, embraced by British empiricists millennia later, and made the core concept in behaviorist cognitive science (McClelland et al., 1986).

The realization that associative bonds do not explain Pavlovian and operant/reinforcement learning also calls into question connectionist approaches to cognitive science (Maurer, 2021). It undermines the claim that Large Language Models do what they do because they work in the same way brains work (Xu & Poo, 2023). Those who make these claims take it for granted that associative bonds are the foundation of biological intelligence. They correctly insist that associative bonds are not symbols: they do not encode anything, nor are they elements in a symbol processing system (Heaven, 2023). Connection weights are the conceptual stuff of Large Language Models but they are not the explanation for associative learning as a biological phenomenon.^4^

## Data Availability

Not applicable.

## Footnotes

1 And this despite the fact that a majority of Gottlieb’s six experiments were done in Rescorla’s lab.

2 Scare quotes are because in these experiments the pigeons used a change-over key to change the color and the associated VI schedule on the main key, the key that on which pecks triggered the feeding opportunities set up by whichever of two VI schedules was operative. A single peck on the change-over key changed the color of the VI key and put the associated VI in force after a short delay.

3 This assumes scientific realism, the belief that minds are embedded in a world in which events unfold in time and space, as they do in the theories of physicists.

4 As a matter of physical fact, the connection weights in an LLM are binary numerals (bit patterns) in the memory of a computer. The sum of the input “weights” represented by the bit patterns in the memory registers is computed by the arithmetic machinery in the CPU to produce the bit patterns that serve as the symbols for the “activation” of the notional neurons in the next layer. The computer’s symbol-processing machinery passes the sum through a nonlinear function to produce a bit pattern that is the symbol for the “signal” that the notional neurons transmit to notional neurons in the next layer. In other words, associations as traditionally conceived, activation-transmitting connections, have no physically realized existence in LLMs—only a conceptual existence. They are simulated using the computer’s symbol processing machinery. Hinton believes that this *simulates* a nonsymbolic biological process, but there is scant evidence that such a process exists (Gallistel & Matzel, 2013). The notional connection weights in LLMs are computed by back-propagation. There is no evidence for this computation in brains and it is wildly implausible because it depends on an omniscient tutor that gives the correct answer after each of the quadrillions of trials. There is, by contrast, abundant behavioral and neurobiological evidence for the computation of counts, durations and rates—computations for which no tutor is required.
